# Association Between Oxidative–Inflammation Biomarkers and Incident Chronic Kidney Disease in People with High Cardiovascular Risk: A Nested Case–Control Study

**DOI:** 10.3390/antiox14080975

**Published:** 2025-08-08

**Authors:** Maria Magdalena Quetglas-Llabrés, Andrés Díaz-López, Cristina Bouzas, Margalida Monserrat-Mesquida, Jordi Salas-Salvadó, Miguel Ruiz-Canela, J. Alfredo Martínez, José Manuel Santos-Lozano, Silvia García, Ramon Estruch, José López-Miranda, Dora Romaguera, Francisco J. Tinahones, Marcos García-Fernández, Sebastián Mas-Fontao, Pilar Matía-Martín, Jesús Vioque, Aurora Bueno, Nancy Babio, Josep A. Tur, Antoni Sureda

**Affiliations:** 1 Research Group on Community Nutrition & Oxidative Stress, University of Balearic Islands-IUNICS, IDISBA & CIBEROBN, Guillem Colom Bldg, Campus, 07122 Palma de Mallorca, Spaincristina.bouzas@uib.es (C.B.); margalida.monserrat@uib.es (M.M.-M.); antoni.sureda@uib.es (A.S.); 2 Health Research Institute of the Balearic Islands (IdISBa), 07120 Palma de Mallorca, Spain; 3 CIBER Fisiopatología de la Obesidad y Nutrición (CIBEROBN), Instituto de Salud Carlos III (ISCIII), 28029 Madrid, Spain; andres.diaz@urv.cat (A.D.-L.);; 4 Department of Basic Medical Sciences, Nutrition and Mental Health (NUTRISAM) Research Group, Universitat Rovira i Virgili (URV), 43204 Reus, Spain; 5 Institut d’Investigació Sanitària Pere Virgili (IISPV), 43201 Reus, Spain; 6 Universitat Rovira i Virgili, Departament de Bioquímica i Biotecnologia, Alimentació, Nutrició, Desenvolupament i Salut Mental (ANUT-DSM), 43201 Reus, Spain; 7 Department of Preventive Medicine and Public Health, University of Navarra, IdiSNA, 31008 Pamplona, Spain; 8 Department of Nutrition, Food Sciences, and Physiology, Center for Nutrition Research, University of Navarra, 31008 Pamplona, Spain; 9 IMDEA Nutrition, CEI UAM + CSIC, 28049 Madrid, Spain; 10 Precision Nutrition and Cardiometabolic Health, University of Valladolid, 47002 Valladolid, Spain; 11 Centre of Medicine and Endocrinology, University of Valladolid, 47002 Valladolid, Spain; 12 Department of Medicine, University of Sevilla, 41009 Sevilla, Spain; 13 Department of Internal Medicine, Institut d’Investigacions Biomèdiques August Pi Sunyer (IDIBAPS), Hospital Clinic, University of Barcelona, 09036 Barcelona, Spain; 14 Department of Internal Medicine, Maimonides Biomedical Research Institute of Cordoba (IMIBIC), Reina Sofia University Hospital, University of Cordoba, 14004 Cordoba, Spain; 15 Department of Endocrinology and Nutrition, Virgen de la Victoria University Hospital, 29010 Malaga, Spain; 16 Instituto de Investigación Biomédica de Málaga (IBIMA), Plataforma Bionand, 29590 Malaga, Spain; 17 CIBER de Epidemiología y Salud Pública (CIBERESP), Instituto de Salud Carlos III, 28029 Madrid, Spain; 18 Research Group on Interactions Gene-Environment and Health (GIIGAS), Institute of Biomedicine (IBIOMED), University of León, 24071 Leon, Spain; 19 Department of Endocrinology and Nutrition, Hospital Fundación Jimenez Díaz, Instituto de Investigaciones Biomédicas IISFJD, University Autonoma, 28040 Madrid, Spain; 20 Department of Endocrinology and Nutrition, Instituto de Investigación Sanitaria Hospital Clínico San Carlos (IdISSC), 28040 Madrid, Spain; 21 CIBER Diabetes y Enfermedades Metabólicas (CIBERDEM), Instituto de Salud Carlos III (ISCIII), 28029 Madrid, Spain; 22 Instituto de Investigación Sanitaria y Biomédica de Alicante (ISABIAL), Universidad Miguel Hernández, 03550 Alicante, Spain; 23 Instituto de Investigación Biosanitaria Ibs-Granada, 18012 Granada, Spain

**Keywords:** kidney dysfunction, inflammation, oxidative stress, metabolic syndrome, glomerular filtration

## Abstract

**Aim**: To assess oxidative–inflammatory biomarker prediction of incident CKD after 1-year follow-up in a population with overweight/obesity and metabolic syndrome. **Methods**: Prospective nested case–control study comprising 117 CKD incident cases and 117 matched controls free of CKD after 1-year follow-up conducted in 55–75-year-old participants. Controls were time-matched 1:1 to cases by intervention group, age (≤65 vs. >65 years), and sex. Serum creatinine (SCr), cystatin C (CyC), and urine albumin-to-creatinine ratio (UACR) were measured at baseline, and CKD Epidemiology Collaboration equations for Caucasians were used to assess SCr, CyC, and CyC-SCr-based estimated Glomerular Filtration Rate (eGFR). Baseline levels of malondialdehyde (MDA), carbonyls, tumour necrosis factor alpha (TNFα), interleukin (IL)-1β, IL-1ra, IL-6, monocyte chemoattractant protein 1 (MCP-1), and leptin were determined from fasting serum samples. An inflammatory-oxidative stress score based on these biomarkers was calculated. Incident CKD was defined by eGFR-SCr <60 mL/min/1.73 m^2^, and/or UACR ≥30 mg/g in the absence of CKD at baseline. **Results**: UACR positively correlated with pro-inflammatory markers (IL-1β; TNFα) and oxidative damage marker (MDA); eGFR-cyC showed negative correlations with IL-1β and IL-1ra, and eGFR-SCr with leptin. The odds ratios (OR; 95% CI) for incident CKD in the highest vs. the lowest tertile of IL-1ra IL-6 and TNFα were (2.22; 1.22–4.04), (7.03; 2.88–17.14), and (3.79; 1.79–8.02), respectively. The inflammatory–oxidative stress score was associated with incident CKD (OR per 1-SD increment: 2.06; 1.49–2.83). **Conclusions**: Inflammatory/oxidative stress is associated with CKD incidence in individuals with high cardiovascular risk, underscoring the importance in identify early inflammation to prevent this disease.

## 1. Introduction

Chronic kidney disease (CKD) is a worldwide health issue, affecting 9–13% of the population, and is associated with substantial morbidity and mortality [1]. In 2023, the International Society of Nephrology estimated that approximately 850 million individuals worldwide were living with CKD, with disadvantaged populations bearing a disproportionately higher risk [2]. In our study population, CKD prevalence is estimated at 15–16%, slightly above the global average [3,4]. This condition is defined by the presence of kidney damage, commonly manifested as proteinuria, a urine albumin-to-creatinine ratio (UACR) ≥ 30 mg/g, an estimated glomerular filtration rate (eGFR) < 60 mL/min/1.73 m^2^, or structural/functional renal abnormalities (e.g., urinary sediment alterations, imaging findings, or histological changes) that persist for at least three months, with or without renal function deterioration, in accordance with international CKD guidelines [5,6]. CKD is more common in men than in women, particularly among individuals over 65 years of age, and its progression is worsened by comorbidities such as metabolic syndrome (MetS). MetS comprises a cluster of interrelated metabolic disturbances, characterized by oxidative stress and a pro-inflammatory state, which increase the risk of hypertension, diabetes, atherosclerosis, and vascular dysfunction, all of which appear to promote renal function impairment [7,8,9].

The kidneys, responsible for waste excretion, electrolyte and acid-base balance, and blood pressure regulation, are highly metabolically active and vulnerable to oxidative stress [10,11]. Mitochondria, essential for kidney physiology, produce reactive oxygen species (ROS) during normal activity. Under conditions such as MetS and ageing, excess ROS production promotes oxidative stress, mitochondrial damage, and reduced cell viability [10]. As such, they are implicated in different pathological situations including renal diseases [12]. Indeed, ROS can directly harm the kidneys’ glomerulus and tubulointerstitial tissue, causing not only the development but also the progression of kidney disease [13]. Previous studies reported inverse correlation between different markers of oxidative stress and eGFR, even in early stages of impaired kidney function [14,15,16]. Several epidemiological studies have reported high levels of biomarkers associated with oxidative stress and inflammation in CKD [17,18,19,20,21]. The pro-oxidant state in MetS also promotes chronic low-grade inflammation and endothelial dysfunction, creating a vicious cycle where kidney damage perpetuates further kidney damage [22].

Macrophages play a critical role in dysregulated immune responses during the progression of renal injury. A shift in macrophage polarization from the anti-inflammatory M2 phenotype to the pro-inflammatory M1 phenotype has been observed to contribute to the pathogenesis and progression of CKD [23]. This polarization shift leads to increased production of pro-inflammatory cytokines such as interleukin (IL)-1 and tumour necrosis factor alpha (TNFα) and ROS, which not only drive tubular epithelial and endothelial damage but also impair capillary integrity, peritubular blood flow, and tight junctions reducing the kidney’s capacity to filter and clear immune mediators [24,25]. As a result, cytokines accumulate in the interstitium, perpetuating leukocyte recruitment and sustaining inflammation via chemokines such as monocyte chemoattractant protein-1 (MCP-1). Additionally, loss of M2-derived anti-inflammatory mediators such as IL-10 further disrupts immunoregulatory feedback, preventing resolution of inflammation [26]. This highlights the critical role of macrophage polarization, cytokine signalling, and kidney function in CKD progression [27]. Moreover, malondialdehyde (MDA) has been shown to promote dysfunctional high-density lipoprotein (HDL) particles [28], contributing to increased cardiovascular morbidity [29]. High levels of carbonyl groups in oxidized proteins have also been observed in various kidney pathologies, serving as good biomarkers to indicate oxidative stress and antioxidant status in kidney disease [30].

While substantial evidence links oxidative stress, inflammation, and CKD, the causal roles of these factors in disease onset remain unclear, particularly in elderly individuals with comorbidities. Consequently, further prospective research would be required to clarify these associations by evaluating inflammatory and oxidative stress markers before CKD onset, while controlling for potentially confounding factors.

Consequently, the current study’s goal was to prospectively assess the association between baseline inflammatory (IL-1β, IL-1ra, IL-6, MCP-1, TNFα, and leptin) and oxidative stress (MDA and carbonyls) biomarkers with new-onset CKD after one year of follow-up in older adults with overweight or obesity and MetS. Given the scarcity of prospective studies evaluating these biomarkers simultaneously in populations with MetS, this study additionally assessed their combined role through a composite inflammatory–oxidative score.

## 2. Methods

### 2.1. Study Design and Participants

Data was analyzed using a prospective case–control design nested within the framework of the Prevención con Dieta Mediterránea (PREDIMED)-Plus trial. The PREDIMED-Plus study is a large, multicentre, parallel-group, randomized clinical trial designed to promote weight loss and reduce major cardiovascular events through an intensive lifestyle intervention [31]. Full details of the protocol and the study design of the PREDIMED-Plus trial (registered on http://www.isrctn.com/ISRCTN89898870, accessed 22 June 2020) are available at https://www.predimedplus.com, accessed 22 June 2020, and were described elsewhere [31,32]. This trial was authorized by the institutional review boards of all participating institutions, and every participant provided written informed permission.

Men and women between the ages of 55 and 75 who were overweight or obese (BMI 27–40 kg/m^2^) and who satisfied at least three MetS criteria [33] without a history of cardi¸; accessed 22 June 2020ovascular disease were eligible to participate. For the present nested case–control study, we additionally excluded individuals with baseline CKD, defined as an eGFR < 60 mL/min/1.73 m^2^ and/or a UACR ≥ 30 mg/g. More specific details of inclusion/exclusion criteria have been extensively reported elsewhere [31,32].

Participants were randomized into two groups: (i) an intensive intervention group, which followed an energy-restricted Mediterranean diet combined with physical activity promotion and behavioural support, and (ii) a control group, which received general recommendations on following a Mediterranean diet but no specific guidance on weight loss or physical activity.

**Figure 1:**
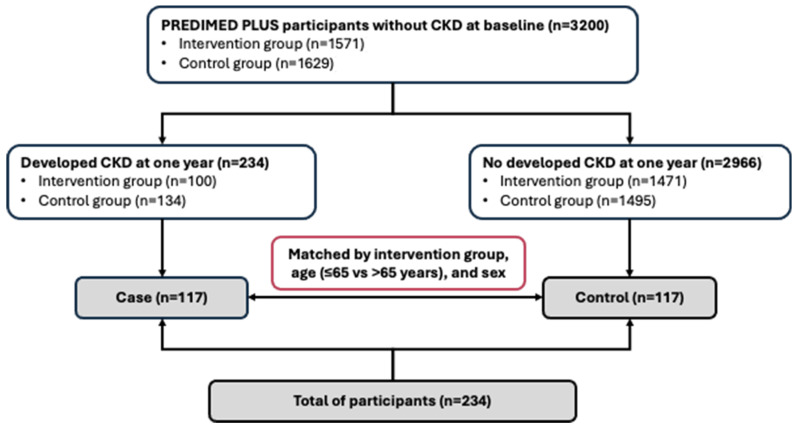
Flowchart of eligible of participants.

A nested case–control study was conducted among non-CKD participants at baseline within the PREDIMED-Plus cohort. Of the 3200 participants with complete kidney function data and free of CKD at baseline, 234 developed new-onset CKD during the first year of follow-up. At baseline, all participants had an eGFRcr ≥ 60 mL/min/1.73 m^2^ (calculated by the CKD-EPI equation) and UACR < 30 mg/g. Over the first year of follow-up, incident CKD was defined as the new occurrence of either an eGFR < 60 mL/min/1.73 m^2^ or a UACR ≥ 30 mg/g, confirmed on two consecutive visits at least three months apart. Participants were recruited between 2014 and 2016 from 20 Spanish research centres involved in the PREDIMED-Plus trial. To ensure feasibility and maintain a balanced sample size for adequate statistical power, we randomly selected 117 incident CKD cases (50% of the total). For each case, a control (n = 117) without CKD at baseline, who remained CKD-free after one year but was still at risk at the time the case was diagnosed, was randomly selected (Figure 1). Cases and controls were individually matched 1:1 based on intervention group, age (≤65 vs. >65 years), and sex.

This study focuses on the association between oxidative stress and inflammatory markers and the risk of developing CKD. Sample size calculations were based on previous studies [34,35] that explored these biomarkers in CKD patients. Assuming a significance level of 0.05 and 90% power in a two-sided test, the smallest sample size needed to find a statistically significant difference of at least 1 pg/mL in IL-6 concentration, with a common standard deviation of 2.15 pg/mL, was estimated at 98 participants per group (98 cases and 98 controls; total = 196). Given that our study includes 117 cases and 117 controls, the sample size is sufficient to assess these associations with robust statistical power.

### 2.2. Evaluation of Kidney Function and Ascertainment of Incident CKD

At baseline and after one-year of follow-up, fasting blood and urine samples were obtained. Serum creatinine (SCr) levels were determined by the enzymatic creatinine assay method (coefficient of variation (CV) < 4%). Baseline serum Cystatin C (cyC), considered a more accurate biomarker of kidney function, was also determined by a Siemens Atellica NEPH 630 (Siemens Healthineers, Marburg, Germany) nephelometer using the Atellica CH CYSC_2 (Siemens Healthcare GmbH) assay (limit of quantitation 0.25 mg/L; CV < 10%). CKD Epidemiology Collaboration equations for Caucasian individuals were used to estimate SCr, cyC, and SCr-cyC-based eGFR [36]. Urinary creatinine and albumin concentrations were determined in single spot urine at baseline using the Jaffé colorimetric method and Bromcresol green albumin method, respectively (CVs, <3.0% and <10%, respectively) and the urinary albumin–creatinine ratio (UACR) was calculated (mg/g). Incident CKD after 1 year of follow-up was defined as progression to CKD stage 3 or greater (eGFR-SCr < 60 mL/min/1.73 m^2^), and/or albuminuria progression (UACR ≥ 30 mg/g) [5].

### 2.3. Serum Inflammatory and Oxidative Stress Biomarker Assessment (Exposure Variables)

A blood sample for each participant was collected from the antecubital vein in suitable vacutainers without anticoagulant after 12 h of overnight fasting and then was centrifugated at 1700× *g* 15 min at 4 °C to obtain serum. Samples were stored at −80 °C.

A total of eight biomarkers, including six inflammatory and two oxidative stress markers, were assessed in serum samples from each participant. TNFα, IL-1, IL-1ra, IL-6, leptin, and MCP-1 were all evaluated at the same time with bead-based multiplexing technology using a Luminex MAGPIX (Millipore) device. Human Custom ProcartaPlex^TM^ (Invitrogen by Thermo Fisher Scientific, Bender MedSystems GmbH, Vienna, Austria) was used for the determinations in accordance with the manufacturer’s instructions.

The levels of malondialdehyde (MDA) were determined using a colorimetric test kit (Sigma-Aldrich Merck^®^, St. Louis, MO, USA) in accordance with the instructions provided by the manufacturer. Glass tubes containing n-methyl-2-phenylindole in a 1:1 methanol/acetonitrile combination were filled with serum samples or standards. After adding HCl (12 N), the samples were incubated at 45 °C for 60 min. A standard curve of known values was then used to compute the MDA concentration after the absorbance was measured at 586 nm.

Total protein levels of the serum samples were determined using a commercial Bradford reagent (Merck Life Science S.L.U., Madrid, Spain) [37]. Protein carbonyl derivatives were measured following the provided guidelines of OxiSelect^TM^ Protein Carbonyl Immunoblot Kit (CELL BIOLABS^®^, San Jose, CA, USA). In total, 10 μg of protein was put into a nitrocellulose membrane and treated with 2,4-dinitrophenylhydrazine (DNPH) in accordance with the dot blot technique (Bio-Rad, Hercules, CA, USA). The primary DNPH-specific antibody (1:1000) was used to incubate the membrane, followed by the secondary goat antirabbit IgG antibody (1:1000). An enhanced chemiluminescence kit (Immun-Star Western C Kit reagent, Bio-Rad Laboratories, Hercules, CA, USA) was then used to generate the immunoblot. Protein carbonyl bands were measured using Quantity One (Bio-Rad Laboratories, Hercules, CA, USA), an image analysis tool.

### 2.4. Assessment of Other Covariables

A generic questionnaire was used to gather baseline data on socio-demographic (age, sex, and educational level) and lifestyle (dietary intake, smoking, physical activity) variables, medicine use, and medical history. Educational level was considered a potential confounder, given its known association with CKD risk through differences in health behaviours, disease awareness, and access to healthcare [38]. The baseline examinations also included the administration of a validated 17-item questionnaire to evaluate adherence to an energy-reduced Mediterranean diet (erMedDiet) [39] and a semiquantitative 145-item validated food frequency questionnaire [40]. Leisure-time physical activity was estimated using the validated Registre Gironí del Cor (REGICOR) questionnaire [41].

Anthropometric measurements (weight and height) and blood pressure (Omron HEM-705CP, Kyoto, Japan) were measured by trained staff following the trial protocol. The body mass index (BMI) was calculated by dividing the weight (kg) by the height squared (m^2^).

Other blood parameters, such as glucose, triglycerides, and low-density lipoprotein cholesterol (LDL-cholesterol), were assessed with the use of standard enzymatic methods. Glycated hemoglobin A1c (HbA1c) measurement was performed using a standardized HPLC according to the Diabetes Control and Complications Trial [42].

### 2.5. Statistical Analysis

The statistical analyses were performed using the Statistical Package for Social Sciences (SPSS v.29, IBM Software Group, Chicago, IL, USA). The PREDIMED-Plus database, updated until August 2021, was used for this study. Data are shown as mean (standard deviation), number (percentage), or mean (95% CI) unless otherwise indicated. Normality of the variables was assessed using the Kolmogorov–Smirnov test. Baseline characteristics between case and control subjects were compared using *t*-tests for the continuous variables with a normal distribution, Mann–Whitney U tests for non-normally distributed continuous variables, and χ^2^ tests for categorical variables. Pearson’s partial correlation analyses was used to assess the correlations between inflammatory (IL-1β, IL-1ra, IL-6, MCP-1, TNFα, and leptin) and oxidative stress (MDA and carbonyls) biomarkers and kidney function markers (UACR, eGFR-SCr, eGFR-cyC, eGFR-SCr cyC) at baseline with adjustment for confounders as intervention group (intervention group, control group), age (years), sex, obesity by BMI (yes, no), education level (primary education, secondary/higher education), physical activity (MET-min/week), diabetes prevalence (yes, no), angiotensin-converting enzyme inhibitors and angiotensin II receptor blockers drugs (yes, no), erMedDiet adherence (continuous), energy intake (kcal/day in quintiles), and smoking status (current, former, never). Subsequently, participants were categorized according to tertiles of inflammatory (IL-1β, IL-1ra, IL-6, MCP-1, TNFα, and leptin) and oxidative stress (MDA and carbonyls) biomarker concentrations based on the distribution of control subjects. A composite inflammatory–oxidative score was created using the eight biomarkers to estimate the overall inflammatory and oxidative profile by summing the values of 1, 2, or 3 points assigned to each tertile of the biomarkers scored positively. The score ranged from 8 to 24 points, with higher scores indicating a higher inflammatory and oxidative profile. We used conditional logistic regression models to estimate the matched odds ratios (ORs) and 95% confidence intervals (CI) for the two highest tertiles compared to the lowest tertile as the reference category in inflammation biomarkers or the overall inflammatory and oxidative profile. Both unadjusted and multivariable-adjusted models were fitted in the conditional logistic regression and correlation analyses. As potential confounders, the same above-mentioned covariates plus one-year changes in body weight (continuous) were included in the correlation analyses, with the exception of the intervention group, age, and sex, as these were used as matching variables. Levels of inflammatory and oxidative stress biomarkers were also modelled as continuous exposures, per 1-SD increase. The *p* for linear trend was calculated, assigning the median value of each category and modelling it as a continuous variable. All statistical tests were 2-tailed, and the significance level was set at *p* < 0.05.

## 3. Results

**Figure 2:**
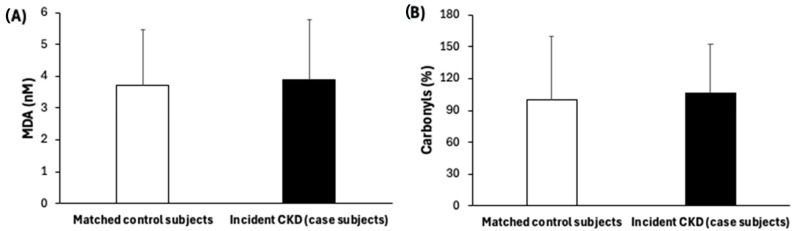
Oxidative stress markers: (**A**) malondialdehyde and (**B**) carbonyl levels in serum. Results are presented as mean (SD). Statistical analysis: Mann–Whitney U test. No significant changes were found, *p* > 0.05.

**Figure 3:**
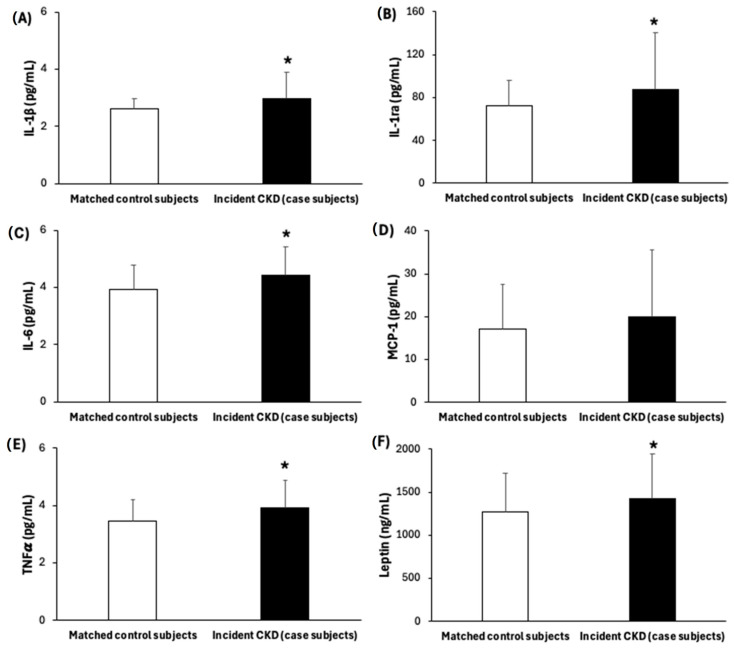
Inflammatory markers: (**A**) IL-1β, (**B**) IL-1ra, (**C**) IL-6, (**D**) MCP-1, (**E**) TNFα, and (**F**) leptin-levels in serum. Results are presented as mean (SD). Statistical analysis: Mann–Whitney U test. * Differences in means between case and control subjects, *p* < 0.05. Abbreviations: IL-1β: interleukin-1β; IL-1ra: interleukin-1ra; IL-6: interleukin-6; MCP-1: monocyte chemoattractant protein 1; TNFα: tumour necrosis factor α.

Table 1 shows the baseline characteristics of participants who developed incident CKD (case subjects, n = 117) and their matched controls (n = 117). Compared to control subjects, individuals who developed CKD after one year had higher baseline BMI values and were more likely to have been diagnosed with T2DM. Furthermore, at baseline they had greater levels of triglycerides, plasma fasting glucose, and HbA1c than their matched control subjects. Baseline kidney function parameters such as UACR, SCr levels, and cyC levels were higher in case subjects than their matched control subjects, while eGFR-SCr, eGFR-cyC, and eGFR-SCr cyC were lower, although these values, despite being less favourable, remained within the normal range and did not meet the clinical criteria for CKD at baseline. Furthermore, higher levels of oxidative stress markers, such as MDA and carbonyls (Figure 2), as well as inflammatory biomarkers, including IL-1β, IL-1ra, IL-6, TNFα, and leptin, were observed in case subjects compared to their matched controls (Figure 3).

The baseline Pearson’s partial correlations between inflammatory and oxidative stress biomarkers and kidney function indicators are shown in Table 2. This analysis was conducted to explore potential associations between these biomarkers and renal function, providing insights into early biological interactions that may contribute to CKD development. Positive correlations were found between UACR and MDA, IL-1β, and TNFα levels in both bivariable analyses and after adjusting for potential confounders, whereas negative correlations were observed between IL-1β and IL-1ra levels and eGFR-cyC values in both bivariable and adjusted analyses. Similarly, eGFR-SCr values exhibited a negative correlation with leptin concentration. Finally, eGFR-SCr cyC was negatively correlated only in the bivariable analysis with IL-1ra and leptin concentrations but not in the multivariate-adjusted model analysis.

The conditional logistic regression analysis showed that high concentrations of various inflammatory biomarkers were closely linked to higher chances of incident CKD (Table 3). Compared with participants in the lowest tertile, participants in the top tertile of IL-1ra (OR; 95% CI: 2.22; 1.22–4.04), IL-6 (OR; 95% CI: 7.03; 2.88–17.14), and TNFα (OR; 95% CI: 3.79; 1.79–8.02) had increased odds of incident CKD. This association was also observed when these inflammatory biomarkers were analyzed as continuous variables, with each 1-SD increase in IL-1ra (OR; 95% CI: 1.57; 1.10–2.24), IL-6 (OR; 95% CI: 1.68; 1.23–2.29), TNFα (OR; 95% CI: 1.80; 1.27–2.56), and IL-1β (OR; 95% CI: 2.03; 1.33–3.08) being significantly associated with CKD risk. Adjustment for potential confounders attenuated the associations in the tertile-based models, with loss of statistical significance for IL-1ra (OR; 95% CI: 2.94, 0.70–12.31) and TNFα (OR; 95% CI: 4.30, 0.86–21.55), although the direction and magnitude of the effects were preserved. In contrast, the associations observed in the continuous models remained statistically significant after adjustment. In the fully adjusted model, participants in the second (OR; 95% CI: 7.89; 2.88–21.63) and third (OR; 95% CI: 11.36; 4.28–30.16) tertiles of the inflammatory–oxidative score had a higher probability of developing incident CKD compared with those in the lowest tertile. This increased risk was also evident when modelling the inflammatory–oxidative score as a continuous variable, where each 1-SD increment was connected to higher odds of incident CKD (OR; 95% CI: 2.06; 1.49–2.83).

Neither when examined as categories nor continuously, in any of the models, were other inflammatory biomarkers like MCP-1 or oxidative stress indicators like MDA and carbonyls linked to incidence CKD.

## 4. Discussion

In this nested case–control study of Mediterranean individuals with overweight/obesity and MetS, higher baseline concentrations of IL-6, IL-1ra, TNFα, and IL-1β were associated with an increased risk of incident CKD after one-year follow-up. After full adjustment, IL-6 remained the only inflammatory biomarker consistently and significantly associated with incident CKD. TNFα and IL-1ra showed positive trends but lost statistical significance in the fully adjusted tertile models, and only TNFα remained significant when modelled as a continuous variable. IL-1β showed significance exclusively in the continuous model. These findings suggest that IL-6 may be the most robust early biomarker of CKD risk in this population, while the associations for IL-1ra, TNFα, and IL-1β appear to be more sensitive to subtle variations in baseline kidney function markers. This reinforces the value of using both continuous and categorical methods when analyzing biomarker data and highlights the importance of interpreting non-significant results with caution when effect directions and magnitudes remain consistent.

We also found significant baseline correlations between oxidative stress (MDA) and inflammatory biomarkers with UACR and eGFR based on serum creatinine and cyC, all of these being kidney function markers. These findings not only suggest that high levels of oxidative and inflammatory parameters may be implicated in the pathogenesis of CKD but also pave the way for potential therapeutic strategies aimed at preserving kidney health in ageing.

Compared to matched controls, case subjects exhibited higher baseline levels of all kidney function parameters, although within normal limits (>60 mL/min/1.73 m^2^), pointing to a possible onset of incipient CKD. In the current study, eGFR was estimated using serum creatinine (eGFR-SCr), cystatin C (eGFR-cyC), and a combination of both markers (eGFR-SCr cyC). The inclusion of both creatinine and cystatin C in eGFR estimation was driven by the need to improve the accuracy of kidney function assessment, as each marker provides complementary information. These findings support previous research highlighting the importance of using different eGFR estimation equations, as each formula has specific strengths and limitations [43]. In all cases, baseline eGFR values were lower in case subjects compared to controls. This highlights the importance of incorporating multiple eGFR estimation methods in clinical practice to enhance the assessment of kidney function and its progression over time.

In the current study, case subjects exhibited significantly higher levels of oxidative stress and inflammatory biomarkers (IL-1β, IL-1ra, IL-6, TNFα, and leptin) than their matched controls. These results are consistent with previous studies reporting altered levels of diverse cytokines and adipokines, including IL-6, TNFα, adiponectin, leptin, and chemerin, in individuals with MetS, reflecting the low-grade inflammatory state characteristic of this disorder [44,45,46].

Notably, the elevated levels of inflammatory markers observed in case subjects at baseline could reinforce the onset of oxidative stress, endothelial dysfunction, heightened sympathetic activity, and, ultimately, lead to alterations in renal function and structure. In line with the current findings, individuals with MetS exhibit low-grade inflammation evidenced by increased secretion of pro-inflammatory factors from adipose tissue, including MCP-1, macrophage migration inhibitory factor, chemokine ligand 5, and macrophage colony-stimulating factor [47]. These pro-inflammatory factors can trigger an inflammatory cascade, culminating in proteinuria and compromised renal function [48]. On the other hand, no differences were observed between cases and controls in the markers of oxidative damage, MDA, and carbonyl groups, suggesting these markers are not early or sensitive enough to distinguish between the two groups with varying risk of developing CKD.

Analyzing the relationship between baseline measurements of UACR, kidney function, eGFR and cystatin C, and a panel of cytokines and oxidative stress biomarkers, we found that higher albuminuria and poorer renal function were associated with elevated serum levels of pro-inflammatory cytokines (IL-1β, IL-1ra, TNFα), lipid oxidation marker (MDA), and leptin. The current findings align with those of the CRIC (Chronic Renal Insufficiency Cohort) study, which also identified a relationship between elevated plasma acute phase proteins (hs-CRP, fibrinogen and albumin) and cytokines (IL-1β, IL-6, and TNFα) with reduced kidney function (cystatin C and eGFR) and albuminuria [20]. The elevated cytokine levels observed in CKD patients can be explained by two complementary mechanisms: (1) the impaired renal filtration, which leads to the accumulation of inflammatory mediators, and (2) the increased production of these biomolecules by affected tissues in response to chronic inflammation and tissue damage. Together, these processes likely drive the persistently high cytokine concentrations characteristic of CKD [20].

The correlation analysis revealed that IL-1β and IL-1ra were the markers most strongly associated with kidney function parameters, followed by leptin. While MDA and TNFα showed positive correlations exclusively with UACR, other markers did not correlate with any kidney parameter. These observations are consistent with previous studies reporting that continuous exposure to damage-associated molecular patterns (DAMPs) induces the release of pro-inflammatory cytokines, such as IL-1β, which triggers pyroptosis, renal inflammation, and ultimately renal dysfunction [49]. This inflammatory response is typically accompanied by an increase in IL-1ra, the natural anti-inflammatory receptor for IL-1β, which often mirrors IL-1β level fluctuations [50].

Despite these observations, other studies have found stronger correlations between cytokines such as IL-6, TNFα, and MCP-1 and renal function decline [51]. In the present analysis, only TNFα was associated with UACR as a marker of renal damage. Similarly, elevated levels of inflammatory cytokines, including IL-6 and TNFα, along with acute-phase proteins like CRP, have been linked to an increased risk of CKD [19,52]. These results highlight the complex and heterogeneous relationship between inflammatory markers and kidney function. While IL-1β and IL-1ra emerged as the most relevant cytokines in this study, the weaker correlations observed for IL-6, TNFα, and MCP-1 suggest that distinct inflammatory profiles may contribute differently to renal damage. This reinforces the need for further research to clarify these differential associations and their clinical implications for CKD progression.

Leptin showed an inverse association with eGFR-SCr in the multivariate adjusted model. Higher leptin levels may be associated with CKD since it is predominantly eliminated through the kidneys, and an impaired renal clearance could contribute to its rise in serum. Additionally, in obese patients, leptin may contribute to obesity-related glomerulopathy, potentially stimulating the renin–angiotensin–aldosterone system and exacerbating proteinuria and CKD [53]. Unlike previous research where patients already suffered from CKD from their recruitment [19,20,52], this study focused on participants without CKD at baseline, thereby highlighting the predictive value of inflammation in early CKD development. These findings emphasize the importance of monitoring inflammatory profiles in MetS populations to mitigate CKD risk.

We found that not all serum inflammatory markers were equally associated with CKD risk. Specifically, IL-6 remained significantly associated with incident CKD in multivariable models, whereas IL-1ra, IL-1β, and TNFα showed only modest or non-significant associations after adjustment. These findings highlight a potential differential contribution of individual cytokines, underlining the importance of comprehensive biomarker profiling in identifying those most predictive of early kidney impairment. However, no significant increased risk was observed for oxidative damage markers. The lack of association for oxidative damage markers may stem from the fact that, at the time of sampling, CKD had not yet developed and thus no significant tissue damage was present. Although pro-inflammatory mediators generally rise simultaneously, the timing and extent of increase vary among individual molecules. One of the most methodologically novel aspects of the current study is the creation of a composite inflammatory–oxidative score, which integrates several biomarkers. This approach enables a more comprehensive assessment of the inflammatory profile and its relationship with CKD, something not commonly found in previous studies [19,20,30]. For this reason, we calculated an inflammation score by integrating the measurements of all pro-inflammatory markers and evidenced a very evident increase in the probability of developing CKD after one year of follow-up. Overall, current results show that participants with declining kidney function after one year had higher baseline inflammation, suggesting that inflammatory mechanisms may play a role in the etiology of CKD.

Participants who developed CKD after a 1-year follow-up had a higher baseline BMI compared to controls, suggesting that overweight and obesity are positively associated with CKD development [54,55]. In addition, previous studies have shown that MetS increases CKD risk independently of BMI [56,57]. The present findings, particularly the differences in BMI and MetS prevalence between cases and controls, suggest a potential synergistic effect between MetS and high BMI in CKD onset and progression, which aligns with prior research [56]. Biochemically, case subjects exhibited higher baseline glucose and HbA1c levels, with 50.4% diagnosed with T2DM. Given that diabetes is a major risk factor for CKD, affecting approximately 40% of individuals with T2DM [58], these metabolic alterations may contribute to an inflammatory and pro-oxidative state, further exacerbating kidney dysfunction. In recognition of the interconnections between CVD, CKD, and T2DM, the American Heart Association has introduced the Cardiovascular-Kidney and Metabolic Syndrome (CKM) concept [59]. A meta-analysis further confirmed that T2DM significantly increases CKD risk, with relatively higher risks of 3.34 in females and 2.84 in males [58]. Additionally, elevated HbA1c levels have been associated with a decline in eGFR in MetS populations, highlighting their role as potential contributors to renal dysfunction [53,60,61]. Importantly, in our study, the associations between inflammatory markers and CKD were observed independently of these metabolic risk factors, as our models were adjusted for lifestyle components (diet, physical activity, smoking), diabetes, obesity, and education level. This suggests that inflammation and oxidative stress may play a direct role in CKD pathogenesis beyond traditional metabolic risk factors, reinforcing the importance of monitoring inflammatory profiles in populations at risk.

## 5. Strengths and Limitations of the Study

The current research has both limitations and strengths. Key strengths include a substantial number of incident cases, a lengthy follow-up period, inclusion of both sexes, and a thorough standardized assessment and control for numerous potential confounding variables. However, several limitations should be acknowledged. First, the nested case–control design may introduce bias in effect estimation, as odds ratios (OR) tend to overestimate the true association when studying frequent diseases. Second, as an observational study, residual confounding cannot be entirely ruled out despite adjustments for multiple covariates. Baseline values of eGFR, UACR, and diabetes prevalence differed between groups; however, all participants were free of CKD at baseline, and these values were within clinically normal ranges. The statistical models were therefore adjusted for these variables to minimize confounding. The original matching strategy based on age, sex, and intervention group was defined a priori in the funding project, which focused specifically on inflammation-related hypotheses. Although the dataset includes clinical variables such as eGFR and UACR for the broader PREDIMED-Plus cohort, biomarker determinations were only carried out for the 234 participants selected within this subproject. These decisions were made to preserve statistical power and feasibility within the funded study scope. Third, the sample size per tertile in stratified analyses was relatively low, which may have reduced statistical power in detecting associations. Fourth, a key limitation inherent to observational studies is the uncertainty regarding the directionality of the association between inflammation and kidney damage. While inflammation is considered a risk factor for CKD, renal dysfunction itself can also trigger inflammatory processes, making it challenging to determine causality. Additionally, the composite inflammatory–oxidative score used in this study was constructed based on internal quartile distribution, which, while suitable for exploratory purposes, may limit its generalizability to other populations. Future studies should aim to establish validated and standardized cut-offs that enable external application and clinical translation. Finally, although this study employed multiple eGFR estimation equations (eGFR-SCr, eGFR-cyC, and eGFR-SCr-cyC) to enhance kidney function assessment, it is important to acknowledge that the accuracy of eGFR estimates can be influenced by non-GFR determinants, including muscle mass, inflammation, and medication use. According to recent CKD guidelines, eGFR-SCr is the standard initial test, but in cases where a more accurate evaluation is required, the addition of cystatin C (eGFR-SCr-cyC) is recommended, as was performed in this study [36]. While our study did not directly assess the clinical impact of different eGFR estimation approaches, the use of multiple equations strengthens the reliability of kidney function assessment in this population. The focus was on individuals at high risk of CVD, which may limit the generalizability of the current findings to the broader population.

## 6. Conclusions

The current study emphasizes the importance of inflammation and oxidative stress in CKD progression, particularly in individuals with MetS. The composite inflammatory–oxidative score, which integrates multiple biomarkers, was associated with CKD risk, suggesting that a multifactorial assessment of inflammatory profiles may be crucial for early disease prevention. Overall, this study provides new insights into the mechanisms underlying CKD development and opens the door to more precise preventive and therapeutic approaches based on the early measurement of inflammatory and metabolic biomarkers. Future work should validate these findings in larger cohorts and assess the incremental predictive value of the composite score when combined with traditional risk factors.

## Figures and Tables

**Table 1:** Sociodemographic, lifestyle characteristics, and kidney function parameters at baseline of study population.

General Characteristics		Matched Control Subjects (n = 117)	Incident CKD (Case Subjects) (n = 117)	*p*-Value *
Age, mean (SD), years		65.7 (4.85)	66.1 (4.57)	0.570
Women, no. (%)		58 (49.6)	58 (49.6)	1.000
Intervention group, no. (%)				1.000
	Lifestyle intervention	53 (45.3)	53 (45.3)	
	Control	64 (54.7)	64 (54.7)	
BMI, mean (SD), kg/m^2^		32.0 (3.51)	33.2 (3.32)	0.006
Obesity (BMI ≥ 30 kg/m^2^), no. (%)		79 (67.5)	91 (77.8)	0.078
Education level, no. (%)				0.043
	Primary education	48 (41.0)	67 (57.3)	
	Secondary/academic or graduate	69 (59.0)	50 (42.7)	
Smoking status, no. (%)				0.913
	Never smoked	53 (45.3)	53 (45.3)	
	Former smoker	49 (41.9)	51 (43.6)	
	Current smoker	15 (12.8)	13 (11.1)	
Physical activity, mean (SD), METs/min/day		376.5 (281.1)	372.2 (355.6)	0.274
erMedDiet score, mean (SD), points		8.77 (2.81)	8.52 (2.86)	0.411
Energy intake, mean (SD), kcal/day		2462 (634)	2326 (609)	0.107
Systolic blood pressure, mean (SD), mmHg		138.9 (15.7)	141.7 (17.3)	0.159
Diastolic blood pressure, mean (SD), mmHg		82.1 (10.7)	80.7 (9.09)	0.278
Hypertension, no. (%)		96 (82.1)	97 (82.9)	0.863
Type 2 diabetes, no. (%) ^a^		23 (19.7)	59 (50.4)	<0.001
Medication use, no. (%)				
	Lipid-lowering drugs	62 (53.0)	67 (57.3)	0.511
	Oral blood glucose-lowering drugs	22 (18.8)	48 (41.0)	<0.001
	Insulin treatment	3 (2.6)	8 (6.8)	0.123
	Antihypertensive drugs	84 (71.8)	94 (80.3)	0.125
	ARBs	35 (29.9)	45 (38.5)	0.168
	ACEis	36 (30.8)	33 (28.2)	0.677
Blood parameters				
Glucose, mean (SD), mg/dL		106.3 (18.4)	120.6 (34.8)	0.002
HbA1c, mean (SD), %		5.91 (0.54)	6.44 (1.05)	<0.001
Triglycerides, mean (SD), mg/dL		137.0 (64.2)	162.8 (82.5)	0.008
LDL-cholesterol, mean (SD), mg/dL		119.2 (31.2)	118.0 (32.3)	0.765
Kidney function parameters				
Uric acid, mean (SD), mg/dL		5.76 (1.31)	5.86 (1.17)	0.535
UACR, mean (SD), mg/g		5.19 (3.37)	12.2 (7.81)	<0.001
Serum Creatinine, mean (SD), mg/dL		0.76 (0.12)	0.81 (0.14)	0.005
CyC, mean (SD), mg/L		1.01 (0.13)	1.09 (0.21)	0.002
eGFR-SCr, mean (SD), mL/min/1.73 m^2^		89.9 (6.46)	85.3 (10.2)	0.003
eGFR-cyC, mean (SD), mL/min/1.73 m^2^		74.2 (12.9)	68.5 (17.0)	0.005
eGFR-SCr cyC, mean (SD), mL/min/1.73 m^2^		81.3 (9.8)	76.4 (13.6)	0.002

Values are reported as mean (SD) for continuous variables and number (%) for categorical variables. The *p*-value for continuous variables was computed using Student’s *t*-test for normally distributed variables or the Mann–Whitney U test for non-normally distributed variables, while chi-square test was used for categorical variables. Statistical significance is indicated by the *p*-values; * *p* < 0.05. Abbreviations: BMI: body mass index; METs: metabolic equivalents (min/day); erMedDiet: energy-restricted Mediterranean diet; ARBs: angiotensin-type 2 receptor blockers; ACEis: angiotensin-converting enzyme inhibitors; HbA1c: Glycated hemoglobin A1c, low-density lipoprotein (LDL-cholesterol); UACR: urine albumin-to-creatinine ratio; CyC: cystatin C; eGFR-SCr: estimated glomerular filtration rate using serum creatinine; eGFR-cyC: estimated glomerular filtration rate using cystatin C; eGFR-SCr cyC: estimated glomerular filtration rate using both serum creatinine and cystatin C; SD: standard deviation. ^a^ HbA1c ≥ 6.5%, antidiabetic drug usage, a history of diabetes, or fasting glucose levels more than 126 mg/dL were all considered indicators of current diabetes.

**Table 2:** Correlation analyses of inflammatory and oxidative stress biomarkers and kidney function markers at baseline.

	Inflammatory Biomarkers							
	MDA, nM	Carbonyls, %	IL-1β, pg/mL	IL-1ra, pg/mL	IL-6, pg/mL	MCP1, pg/mL	TNFα, pg/mL	Leptin, ng/mL
Kidney Function Markers	r	r	r	r	r	r	r	r
Bivariable analysis								
UACR, mg/g	0.14 *	0.04	0.21 **	0.03	0.10	0.10	0.20 **	0.10
eGFR-SCr, mL/min/1.73 m^2^	0.00	0.02	−0.09	−0.06	−0.11	−0.12	−0.03	−0.18 **
eGFR-cyC, mL/min/1.73 m^2^	−0.01	−0.08	−0.14 *	−0.18 **	−0.11	−0.05	−0.05	−0.11
eGFR-SCr cyC, mL/min/1.73 m^2^	0.00	−0.03	−0.11	−0.17 *	−0.10	−0.09	−0.06	−0.15 *
Multivariate-adjusted models *								
UACR, mg/g	0.16 *	0.01	0.20 *	0.03	0.11	0.09	0.19 *	0.09
eGFR-SCr, mL/min/1.73 m^2^	0.02	−0.01	−0.07	−0.06	−0.07	−0.09	−0.06	−0.17 *
eGFR-cyC, mL/min/1.73 m^2^	0.02	−0.07	−0.14 *	−0.14 *	−0.09	−0.01	−0.08	−0.08
eGFR-SCr cyC, mL/min/1.73 m^2^	0.04	−0.03	−0.12	−0.12	−0.07	−0.06	−0.11	−0.12

Data are Pearson’s correlation coefficients. Adjusted for intervention group (intensive lifestyle intervention group or control group), age (≤65 vs. >65 years), sex, obesity at baseline, education level (primary, secondary education, graduate), physical activity (MET·min/day), diabetes prevalence (yes/no), angiotensin-converting enzyme inhibitors and angiotensin II receptor blockers drugs (yes/no), energy restricted Mediterranean Diet adherence (continuous), energy intake (kcal/day in quintiles), and smoking status (current, former, never). Statistical significance is indicated by * *p*-value < 0.05 and ** *p*-value < 0.01.

**Table 3:** Odds ratios and 95% CI for incident CKD according to tertile of inflammatory and oxidative stress biomarkers and inflammatory–oxidative score.

			Tertile of Oxidative Stress and Inflammatory Biomarkers			
		Continuous (per 1-SD Increase)	1st Tertile	2nd Tertile OR (95% CI)	3rd Tertile OR (95% CI)	*p* for Trend
MDA, nM			≤2.77	2.77–3.73	>3.73	
	No. Case (%)/control (%)	117(50)/117(50)	31 (13.2)/41 (17.5)	37 (15.8)/38 (16.2)	49 (20.9)/38 (16.2)	
	Crude (matched ^a^) model	1.10 (0.85–1.41)	1.00 ref	1.29 (0.67–2.46)	1.64 (0.89–3.02)	0.121
	Multivariable model †	1.03 (0.53–2.02)	1.00 ref	1.70 (0.49–5.86)	0.90 (0.24–3.44)	0.646
Carbonyls, %			≤70.68	70.68–107.17	≥107.17	
	No. Case (%)/control (%)	117(50)/117(50)	20 (8.5)/39 (16.7)	56 (23.9)/39 (16.7)	41 (17.5)/39 (16.7)	
	Crude (matched ^a^) model	1.12 (0.86–1.45)	1.00 ref	2.70 (1.36–5.35) **	2.04 (1.03–4.06) *	0.253
	Multivariable model †	0.87 (0.45–1.66)	1.00 ref	3.99 (0.89–17.9)	2.95 (0.64–13.7)	0.655
IL-1β, pg/mL			≤2.42	2.42–2.57	>2.57	
	No. Case (%)/control (%)	117(50)/117(50)	29 (12.4)/38 (16.2)	32 (13.7)/39 (16.7)	56 (23.9)/40 (17.1)	
	Crude (matched ^a^) model	2.03 (1.33–3.08) **	1.00 ref	1.06 (0.54–2.08)	1.85 (0.97–3.53)	0.038
	Multivariable model †	2.84 (1.04–7.74) *	1.00 ref	1.28 (0.35–4.75)	2.94 (0.70–12.31)	0.111
IL-1ra, pg/mL			≤59.23	59.23–69.77	>69.77	
	No. Case (%)/control (%)	117(50)/117(50)	29 (12.4)/49 (20.9)	31 (13.2)/28 (12.0)	57 (24.4)/40 (17.1)	
	Crude (matched ^a^) model	1.57 (1.10–2.24) *	1.00 ref	1.88 (0.92–3.85)	2.22 (1.22–4.04) **	0.022
	Multivariable model †	2.15 (1.13–4.11) *	1.00 ref	4.27 (0.93–19.3)	4.27 (0.93–19.7)	0.089
IL-6, pg/mL			≤3.57	3.57–3.81	>3.81	
	No. Case (%)/control (%)	117(50)/117(50)	8 (3.4)/39 (16.7)	42 (17.9)/41 (17.5)	67 (28.6)/37 (15.8)	
	Crude (matched ^a^) model	1.68 (1.23–2.29) **	1.00 ref	4.86 (1.92–12.33) **	7.03 (2.88–17.14) **	<0.001
	Multivariable model †	2.09 (1.11–3.95) *	1.00 ref	7.32 (1.03–52.09) *	7.94 (1.45–43.6) *	0.065
MCP1, pg/mL			≤10.3	10.3–19.9	>19.9	
	No. Case (%)/control (%)	117(50)/117(50)	36 (15.4)/40 (17.1)	38 (16.2)/38 (16.2)	43 (18.4)/39 (16.7)	
	Crude (matched ^a^) model	1.26 (0.96–1.67)	1.00 ref	1.10 (0.60–2.00)	1.22 (0.66–2.28)	0.530
	Multivariable model †	1.46 (0.75–2.85)	1.00 ref	0.90 (0.24–3.42)	0.90 (0.23–3.54)	0.899
TNFα, pg/mL			≤3.17	3.17–3.52	>3.52	
	No. Case (%)/control (%)	117(50)/117(50)	19 (8.1)/39 (16.7)	30 (12.8)/38 (16.2)	68 (29.1)/40 (17.1)	
	Crude (matched ^a^) model	1.80 (1.27–2.56) **	1.00 ref	1.87 (0.87–4.05)	3.79 (1.79–8.02) **	<0.001
	Multivariable model †	2.27 (1.01–5.09) *	1.00 ref	2.05 (0.37–11.3)	4.30 (0.86–21.55)	0.069
Leptin, ng/mL			≤1101.4	1101.4–1518.11	>1518.11 ng/mL	
	No. Case (%)/control (%)	117(50)/117(50)	50 (21.4)/39 (16.7)	36 (15.4)/40 (17.1)	31 (13.2)/38 (16.2)	
	Crude (matched ^a^) model	1.38 (1.05–1.82) *	1.00 ref	0.72 (0.40–1.29)	0.65 (0.35–1.21)	0.163
	Multivariable model †	1.97 (0.91–4.28)	1.00 ref	2.09 (0.52–8.49)	3.39 (0.59–19.52)	0.170
Inflammatory–oxidative score, mean (SD), point			13.8 (1.30), ≤15	16.5 (0.51), 15–17	19.7 (1.58), >17	
	No. Case (%)/control (%)	117(50)/117(50)	16 (6.8)/60 (25.6)	40 (17.1)/29 (12.4)	61 (26.1)/28 (12.0)	
	Crude (matched ^a^) model	2.06 (1.49–2.83) **	1.00 ref	7.89 (2.88–21.63) **	11.36 (4.28–30.16) **	<0.001
	Multivariable model †	2.14 (1.13–4.07) *	1.00 ref	41.66 (2.47–703.91) **	22.39 (2.16–232.51) **	0.011

Results are expressed as ORs (95% CI) for CKD from conditional logistic regressions. ^a^ Matched for intervention group (intensive lifestyle intervention group or control group), age (≤65 vs. >65 years), and sex. † Multivariable model was adjusted for obesity (yes/no), education level (primary, secondary education, graduate), physical activity (MET·min/day), diabetes prevalence (yes/no), angiotensin-converting enzyme inhibitors and angiotensin II receptor blockers drugs (yes/no), energy restricted Mediterranean Diet adherence (continuous), energy intake (kcal/day in quintiles), smoking status (current, former, never), one-year changes in body weight (continuous), and baseline kidney function parameters including eGFR-SCr and UACR. Statistical significance is indicated by * *p*-value < 0.05 and ** *p*-value < 0.01. The *p*-value for trend based on the tertile of inflammatory biomarkers as a continuous variable.

## Data Availability

There are restrictions on the availability of data for this trial, due to the signed consent agreements around data sharing, which only allow access to external researchers for studies following the project purposes. Researchers wishing to access the trial data used in this study can make a request to pep.tur@uib.es.

## References

[1] Bikbov B., Purcell C.A., Levey A.S., Smith M., Abdoli A., Abebe M., Adebayo O.M., Afarideh M., Agarwal S.K., Agudelo-Botero M. (2020). Global, regional, and national burden of chronic kidney disease, 1990–2017: A systematic analysis for the Global Burden of Disease Study 2017. Lancet.

[2] Jager K.J., Kovesdy C., Langham R., Rosenberg M., Jha V., Zoccali C. (2019). A single number for advocacy and communication—Worldwide more than 850 million individuals have kidney diseases. Kidney Int..

[3] Llisterri J.L., Micó-Pérez R.M., Velilla-Zancada S., Rodríguez-Roca G.C., Prieto-Díaz M.Á., Martín-Sánchez V., Barquilla A., Polo-García J., Segura-Fragoso A., Cinza-Sanjurjo S. (2021). Prevalence of chronic kidney disease and associated factors in the Spanish population attended in primary care: Results of the IBERICAN study. Med. Clin..

[4] Gorostidi M., Sánchez-Martínez M., Ruilope L.M., Graciani A., de la Cruz J.J., Santamaría R., del Pino M.D., Guallar-Castillón P., de Álvaro F., Rodríguez-Artalejo F. (2018). Prevalencia de enfermedad renal crónica en España: Impacto de la acumulación de factores de riesgo cardiovascular. Nefrología.

[5] Webster A.C., Nagler E.V., Morton R.L., Masson P. (2017). Chronic Kidney Disease. Lancet.

[6] Fernández López P., Romero Lerma Á. (2023). Consideraciones sobre el consenso español multisociedad de manejo de la enfermedad renal crónica. Med. Fam. Semer..

[7] Vona R., Gambardella L., Cittadini C., Straface E., Pietraforte D. (2019). Biomarkers of Oxidative Stress in Metabolic Syndrome and Associated Diseases. Oxid. Med. Cell Longev..

[8] Lerman L.O., Lerman A. (2011). The metabolic syndrome and early kidney disease: Another link in the chain?. Rev. Esp. Cardiol..

[9] Silveira Rossi J.L., Barbalho S.M., Reverete de Araujo R., Bechara M.D., Sloan K.P., Sloan L.A. (2022). Metabolic syndrome and cardiovascular diseases: Going beyond traditional risk factors. Diabetes. Metab. Res. Rev..

[10] Bhargava P., Schnellmann R.G. (2017). Mitochondrial energetics in the kidney. Nat. Rev. Nephrol..

[11] Sureshbabu A., Ryter S.W., Choi M.E. (2015). Oxidative stress and autophagy: Crucial modulators of kidney injury. Redox Biol..

[12] Cachofeiro V., Goicochea M., de Vinuesa S.G., Oubiña P., Lahera V., Luño J. (2008). Oxidative stress and inflammation, a link between chronic kidney disease and cardiovascular disease. Kidney Int..

[13] Piko N., Bevc S., Hojs R., Ekart R. (2023). The Role of Oxidative Stress in Kidney Injury. Antioxidants.

[14] Tbahriti H.F., Kaddous A., Bouchenak M., Mekki K. (2013). Effect of different stages of chronic kidney disease and renal replacement therapies on oxidant-antioxidant balance in uremic patients. Biochem. Res. Int..

[15] Ishizaka Y., Yamakado M., Toda A., Tani M., Ishizaka N. (2013). Relationship between estimated glomerular filtration rate, albuminuria, and oxidant status in the Japanese population. BMC Nephrol..

[16] Cottone S., Mule G., Guarneri M., Palermo A., Lorito M.C., Riccobene R., Arsena R., Vaccaro F., Vadala A., Nardi E. (2008). Endothelin-1 and F2-isoprostane relate to and predict renal dysfunction in hypertensive patients. Nephrol. Dial. Transplant..

[17] Xu G., Luo K., Liu H., Huang T., Fang X., Tu W. (2015). The progress of inflammation and oxidative stress in patients with chronic kidney disease. Ren. Fail..

[18] Neelofar K., Arif Z., Arafat M.Y., Alam K., Ahmad J. (2019). A study on correlation between oxidative stress parameters and inflammatory markers in type 2 diabetic patients with kidney dysfunction in north Indian population. J. Cell Biochem..

[19] Amdur R.L., Feldman H.I., Gupta J., Yang W., Kanetsky P., Shlipak M., Rahman M., Lash J.P., Townsend R.R., Ojo A. (2016). Inflammation and Progression of CKD: The CRIC Study. Clin. J. Am. Soc. Nephrol..

[20] Gupta J., Mitra N., Kanetsky P.A., Devaney J., Wing M.R., Reilly M., Shah V.O., Balakrishnan V.S., Guzman N.J., Girndt M. (2012). Association between Albuminuria, Kidney Function, and Inflammatory Biomarker Profile in CKD in CRIC. Clin. J. Am. Soc. Nephrol..

[21] Upadhyay A., Larson M.G., Guo C.-Y., Vasan R.S., Lipinska I., O’Donnell C.J., Kathiresan S., Meigs J.B., Keaney J.F., Rong J. (2011). Inflammation, kidney function and albuminuria in the Framingham Offspring cohort. Nephrol. Dial. Transplant..

[22] Duni A., Liakopoulos V., Roumeliotis S., Peschos D., Dounousi E. (2019). Oxidative Stress in the Pathogenesis and Evolution of Chronic Kidney Disease: Untangling Ariadne’s Thread. Int. J. Mol. Sci..

[23] Lee H., Fessler M.B., Qu P., Heymann J., Kopp J.B. (2020). Macrophage polarization in innate immune responses contributing to pathogenesis of chronic kidney disease. BMC Nephrol..

[24] Dousdampanis P., Aggeletopoulou I., Mouzaki A. (2025). The role of M1/M2 macrophage polarization in the pathogenesis of obesity-related kidney disease and related pathologies. Front. Immunol..

[25] Li G., Yang H., Zhang D., Zhang Y., Liu B., Wang Y., Zhou H., Xu Z.-X., Wang Y. (2024). The role of macrophages in fibrosis of chronic kidney disease. Biomed. Pharmacother..

[26] Brennan E., Kantharidis P., Cooper M.E., Godson C. (2021). Pro-resolving lipid mediators: Regulators of inflammation, metabolism and kidney function. Nat. Rev. Nephrol..

[27] Kadatane S.P., Satariano M., Massey M., Mongan K., Raina R. (2023). The Role of Inflammation in CKD. Cells.

[28] Boaz M., Matas Z., Biro A., Katzir Z., Green M., Fainaru M., Smetana S. (1999). Serum malondialdehyde and prevalent cardiovascular disease in hemodialysis. Kidney Int..

[29] Shao B., Pennathur S., Pagani I., Oda M.N., Witztum J.L., Oram J.F., Heinecke J.W. (2010). Modifying apolipoprotein A-I by malondialdehyde, but not by an array of other reactive carbonyls, blocks cholesterol efflux by the ABCA1 pathway. J. Biol. Chem..

[30] Tucker P.S., Dalbo V.J., Han T., Kingsley M.I. (2013). Clinical and research markers of oxidative stress in chronic kidney disease. Biomarkers.

[31] Martínez-González M.A., Buil-Cosiales P., Corella D., Bulló M., Fitó M., Vioque J., Romaguera D., Martínez J.A., Wärnberg J., López-Miranda J. (2019). Cohort Profile: Design and methods of the PREDIMED-Plus randomized trial. Int. J. Epidemiol..

[32] Salas-Salvadó J., Díaz-López A., Ruiz-Canela M., Basora J., Fitó M., Corella D., Serra-Majem L., Wärnberg J., Romaguera D., Estruch R. (2019). Effect of a lifestyle intervention program with energy-restricted Mediterranean diet and exercise on weight loss and cardiovascular risk factors: One-year results of the PREDIMED-Plus trial. Diabetes Care.

[33] Alberti K.G.M.M., Eckel R.H., Grundy S.M., Zimmet P.Z., Cleeman J.I., Donato K.A., Fruchart J.-C., James W.P.T., Loria C.M., Smith S.C. (2009). Harmonizing the Metabolic Syndrome. Circulation.

[34] Oberg B.P., McMenamin E., Lucas F.L., McMonagle E., Morrow J., Ikizler T.A.L.P., Himmelfarb J. (2004). Increased prevalence of oxidant stress and inflammation in patients with moderate to severe chronic kidney disease. Kidney Int..

[35] Rossi M., Campbell K.L., Johnson D.W., Stanton T., Vesey D.A., Coombes J.S., Weston K.S., Hawley C.M., McWhinney B.C., Ungerer J.P.J. (2014). Protein-bound Uremic Toxins, Inflammation and Oxidative Stress: A Cross-sectional Study in Stage 3–4 Chronic Kidney Disease. Arch. Med. Res..

[36] Stevens P.E., Ahmed S.B., Carrero J.J., Foster B., Francis A., Hall R.K., Herrington W.G., Hill G., Inker L.A., Kazancıoğlu R. (2024). KDIGO 2024 Clinical Practice Guideline for the Evaluation and Management of Chronic Kidney Disease. Kidney Int..

[37] MM B. (1976). A rapid and sensitive method for the quantitation of microgram quantities of protein utilizing the principle of protein-dye binding. Anal. Biochem..

[38] Vart P., Gansevoort R.T., Coresh J., Reijneveld S.A., Bültmann U. (2013). Socioeconomic Measures and CKD in the United States and The Netherlands. Clin. J. Am. Soc. Nephrol..

[39] Schröder H., Zomeño M.D., Martínez-González M.A., Salas-Salvadó J., Corella D., Vioque J., Romaguera D., Martínez J.A., Tinahones F.J., Miranda J.L. (2021). Validity of the energy-restricted Mediterranean Diet Adherence Screener. Clin. Nutr..

[40] Schröder H., Covas M.I., Marrugat J., Vila J., Pena A., Alcántara M., Masiá R. (2001). Use of a three-day estimated food record, a 72-h recall and a food-frequency questionnaire for dietary assessment in a Mediterranean Spanish population. Clin. Nutr..

[41] Molina L., Sarmiento M., Peñafiel J., Donaire D., Garcia-Aymerich J., Gomez M., Ble M., Ruiz S., Frances A., Schröder H. (2017). Validation of the Regicor Short Physical Activity Questionnaire for the Adult Population. PLoS ONE.

[42] (2009). International Expert Committee Report on the Role of the A1C Assay in the Diagnosis of Diabetes. Diabetes Care.

[43] Khalid U.B., Haroon Z.H., Aamir M., Ain Q.U., Mansoor K., Jaffar S.R. (2020). Comparison of Estimated Glomerular Filtration Rate with Both Serum Creatinine and Cystatin C (eGFRcr-cys) versus Single Analyte (eGFRcr or eGFRcys) Using CKD-EPI and MDRD Equations in Tertiary Care Hospital Settings. J. Coll. Physicians Surg. Pak..

[44] Monserrat-Mesquida M., Quetglas-Llabrés M., Capó X., Bouzas C., Mateos D., Pons A., Tur J.A., Sureda A. (2020). Metabolic Syndrome Is Associated with Oxidative Stress and Proinflammatory State. Antioxidants.

[45] López-Jaramillo P., Gómez-Arbeláez D., López-López J., López-López C., Martínez-Ortega J., Gómez-Rodríguez A., Triana-Cubillos S. (2014). The role of leptin/adiponectin ratio in metabolic syndrome and diabetes. Horm. Mol. Biol. Clin. Investig..

[46] Jialal I. (2023). Chemerin levels in metabolic syndrome: A promising biomarker. Arch. Physiol. Biochem..

[47] Engin A.B. (2024). Message Transmission Between Adipocyte and Macrophage in Obesity. Obesity and Lipotoxicity.

[48] Lin L., Tan W., Pan X., Tian E., Wu Z., Yang J. (2022). Metabolic Syndrome-Related Kidney Injury: A Review and Update. Front. Endocrinol..

[49] Islamuddin M., Qin X. (2024). Renal macrophages and NLRP3 inflammasomes in kidney diseases and therapeutics. Cell Death Discov..

[50] Balakrishnan V.S., Schmid C.H., Jaber B.L., Natov S.N., King A.J., Pereira B.J.G. (2000). Interleukin-1 Receptor Antagonist Synthesis by Peripheral Blood Mononuclear Cells. J. Am. Soc. Nephrol..

[51] Batal I., De Serres S.A., Mfarrej B.G., Grafals M., Pinkus G.S., Kalra A., Weins A., Bijol V., Rennke H.G., Guleria I. (2014). Glomerular Inflammation Correlates With Endothelial Injury and With IL-6 and IL-1β Secretion in the Peripheral Blood. Transplantation.

[52] Shankar A., Sun L., Klein B.E.K., Lee K.E., Muntner P., Nieto Javier F., Tsai M.Y., Cruickshanks K.J., Schubert C.R., Brazy P.C. (2011). Markers of inflammation predict the long-term risk of developing chronic kidney disease: A population-based cohort study. Kidney Int..

[53] Kawamoto R., Akase T., Ninomiya D., Kumagi T., Kikuchi A. (2019). Metabolic syndrome is a predictor of decreased renal function among community-dwelling middle-aged and elderly Japanese. Int. Urol. Nephrol..

[54] Foster M.C., Hwang S.-J., Larson M.G., Lichtman J.H., Parikh N.I., Vasan R.S., Levy D., Fox C.S. (2008). Overweight, Obesity, and the Development of Stage 3 CKD: The Framingham Heart Study. Am. J. Kidney Dis..

[55] Jung C.H., Lee M.J., Kang Y.M., Hwang J.Y., Kim E.H., Park J.-Y., Kim H.-K., Lee W.J. (2015). The risk of chronic kidney disease in a metabolically healthy obese population. Kidney Int..

[56] Moeinzadeh F., Rouhani M.H., Seirafian S., Vahdat S., Mortazavi M., Clark C.C.T., Shahdadian F. (2023). Metabolic health status and renal disorders: A cross-sectional study. Sci. Rep..

[57] Chen H.-Y., Lu F.-H., Chang C.-J., Wang R.-S., Yang Y.-C., Chang Y.-F., Wu J.-S. (2020). Metabolic abnormalities, but not obesity per se, associated with chronic kidney disease in a Taiwanese population. Nutr. Metab. Cardiovasc. Dis..

[58] Shen Y., Cai R., Sun J., Dong X., Huang R., Tian S., Wang S. (2017). Diabetes mellitus as a risk factor for incident chronic kidney disease and end-stage renal disease in women compared with men: A systematic review and meta-analysis. Endocrine.

[59] Ndumele C.E., Rangaswami J., Chow S.L., Neeland I.J., Tuttle K.R., Khan S.S., Coresh J., Mathew R.O., Baker-Smith C.M., Carnethon M.R. (2023). Cardiovascular-Kidney-Metabolic Health: A Presidential Advisory From the American Heart Association. Circulation.

[60] Navaneethan S.D., Schold J.D., Kirwan J.P., Arrigain S., Jolly S.E., Poggio E.D., Beddhu S., Nally J.V. (2013). Metabolic Syndrome, ESRD, and Death in CKD. Clin. J. Am. Soc. Nephrol..

[61] Rizk J.G., Hsiung J.-T., Arif Y., Hashemi L., Sumida K., Kovesdy C.P., Kalantar-Zadeh K., Streja E. (2023). Triglycerides and Renal Outcomes According to Albuminuria and in Consideration of Other Metabolic Syndrome Components in Diabetic US Veterans. Am. J. Nephrol..

